# Effect of Mild Conditions on PVA-Based Theta Gel Preparation: Thermal and Rheological Characterization

**DOI:** 10.3390/ijms252212039

**Published:** 2024-11-09

**Authors:** Simone Pepi, Luigi Talarico, Gemma Leone, Claudia Bonechi, Marco Consumi, Amedeo Boldrini, Alessia Lauro, Agnese Magnani, Claudio Rossi

**Affiliations:** 1 Department of Biotechnology, Chemistry and Pharmacy, University of Siena, Via A. Moro 2, 53100 Siena, Italy; simone.pepi@unisi.it (S.P.); luigi.talarico@unisi.it (L.T.); claudia.bonechi@unisi.it (C.B.); marco.consumi@unisi.it (M.C.); amedeo.boldrini@student.unisi.it (A.B.); claudio.rossi@unisi.it (C.R.); 2 National Interuniversity Consortium of Materials Science and Technology (INSTM), Via G. Giusti 9, 50121 Firenze, Italy; 3 Centre for Colloid and Surface Science (CSGI), Via della Lastruccia 3, Sesto Fiorentino (FI), 50019 Florence, Italy

**Keywords:** PVA-PEG theta gel, basic pH, room temperature, aqueous environment, thermal analysis, mechanical behavior, swelling, statistical analysis

## Abstract

Polyvinyl alcohol (PVA), possessing a strong ability to form hydrogels, has been widely used for various pharmaceutical and biomedical applications. In particular, the use of PVA-PEG in the form of theta gels for altered cartilage treatment has attracted an enormous amount of attention in the last 20 years. In this paper, we prepared 42 PVA-PEG in the form of theta gels at room temperature in an aqueous environment, testing the crystallization occurrence at basic pH (10 or 12). Using a statistical approach, the effect of PEG molecular weight, PVA molecular weight and alkaline pH values on water content and mechanical performance was evaluated. The used procedure permitted the theta gels to maintain swelling properties comparable to those of human cartilage, from 60% to 85%, with both polymers having the same influence. PEG MW mainly affected the hydrophilic properties, whereas the thermal properties were mostly influenced by the PVA. The shear and compression mechanical behavior of the produced materials were affected by both the polymers’ MWs. The sample obtained using PVA 125 kDa with PEG 20 kDa as a porogen appeared to be the most suitable one for cartilage disease treatment, as it had an equilibrium shear modulus in the range of 50–250 kPa, close to that of native articular cartilage, as well as optimal mechanical response under compression along the entire analyzed frequency range with a mean value of 0.12 MPa and a coefficient of friction (COF) which remained under 0.10 for all the tested sliding speeds (mm/s).

## 1. Introduction

Polyvinyl alcohol (PVA), a synthetic thermoplastic polymer that is soluble in water and in organic solvents, depending on its molecular weight, is usually prepared by polymerization of vinyl acetate (VAc), followed by partial or complete hydrolysis [1]. The chain length and degree of hydrolysis affect its solubility and properties in water [2]. Indeed, during the shift from low molecular weight (25–35 kDa) to ultrahigh molecular weight (250–300 kDa), a drastic decrease in water solubility can be observed [3]. Similarly, optimum water solubility occurs at 87–89% hydrolysis, whereas at higher hydrolysis degrees, PVA shows a high tendency to form gels through hydrogen-bonding interactions [4]. Its ability to form films and hydrogels, its nontoxicity, its water-solubility, its lack of carcinogenicity, its hydrophilicity, and its good compatibility with human tissues and fluids make PVA of great interest for various pharmaceutical and biomedical applications. Several examples of PVA hydrogels used as drug-delivery systems [5,6], soft tissue substitutes or wound dressing materials have been reported [7,8,9,10]. Among them, the use of PVA for altered cartilage treatment has gained enormous attention in the last 20 years [11,12,13,14,15]. Nevertheless, native PVA hydrogels have low mechanical resistance, and thus, they still face several limitations with regard to their potential widespread use for cartilage repair. Several approaches have been explored to increase the mechanical resistance of PVA hydrogels, from chemical crosslinking using different agents, such as glutaraldehyde [16], boric and boronic acids [17], sodium tri-metaphosphate (STMP) alone [18] or in addition to nanohydroxyapatite [19], to different physical routes, such as cyclic freeze–thawing [20,21]. Starting with the work of Ruberti and Braithwaite [22] the use of theta gel method has been applied to improve the crystallinity and mechanical properties of physically crosslinked PVA hydrogels. The theta gel formation mechanism consists of physical phase separation of a high-molecular-weight polymer solution (e.g., PVA) using a gelling agent (e.g., low-molecular-weight poly(ethylene glycol) (PEG)) that is able to reduce the quality of the solvent, forcing the high-molecular-weight polymer to phase separate and crystallize, forming a physically crosslinked hydrogel network [23]. Polyethylene glycol (PEG) is one of the most used porogen/gelling substances, since it is water-soluble and compatible with almost all polymers [24]. Moreover, being obtainable with variable molecular weights, it can drastically affect phase behaviors and the resulting structures. Theta gels obtained by mixing PVA and PEG for cartilage regeneration have been prepared using different procedures [25,26,27]. Chunming et al., in their study on hybrid PVA-PEG hydrogels, found that introducing PEG to PVA hydrogels endowed the hydrogels, whereas the addition of new chemical crosslinking points due to irradiation provoked the loss of that property [28]. Zhang et al. produced a low-friction, self-healing and high-toughness dual-network composite hydrogel using a simple mixture of PVA (10 wt%) and PEG (30 wt%) [29]. Similarly, a significant improvement in the mechanical response of PVA/PEG composite hydrogels was obtained by a one-step physical crosslinking method further modified by organic solvent immersion [30]. Charron et al. used both freeze–thawing and the theta gel method to further improve the mechanical resistance of PVA [23]. The effect of PEG concentrations [31] and molecular weight [28] on mechanical properties have also been analyzed. Bodugoz-Senturk et al. found that increasing PEG’s molecular weight resulted in a mechanically stronger gel [32]. All these strategies are aimed at promoting crystallization processes that can result in a reduction in water content. Consequently, other routes to find an adequate increase in the crystallinity without losing the correct water content, for the foreseen application, have been exploited in recent years. Huang et al. tested a deprotonation–complexation reprotonation strategy to create strong and tough PVA-based hydrogels using DMSO as a solvent and found that alkali content manipulated the number of crystal nuclei by providing nucleation sites [33]. A similarly strong PVA hydrogel was obtained by Darabi et al. [34] using a high concentration of alkaline metal hydroxide.

In this scenario, we prepared 42 different physical PVA-PEG-based theta gels with the aim of evaluating, using a statistical approach, the effect of not only PEG molecular weight but also PVA molecular weight and alkaline pH values on water content and mechanical performance, exploiting the possibility of obtaining theta gels working at room temperature in a water environment and testing the crystallization occurrence at basic pH (10 or 12).

## 2. Results and Discussion

### 2.1. PVA Framework Synthesis

PVA belonging to three molecular weight classes (low MW: 25–35 kDa; medium: 40–85 kDa; high: 120–220 kDa) and two hydrolysis degree classes (high: 98–99%; low: 87–89%) was used to prepare hydrogels. An identical concentration of both polymers was selected to evaluate only the effect of the polymers’ molecular weight and basic pH values. A concentration of 10% *w*/*w* (molar ratio 1:1) was chosen to ensure a homogeneous aqueous solution while promoting adequate phase separation. Liu et al. found that PEG concentrations above 10% resulted in weak phase separation [31], while Zhang et al. reported that excessive PEG content led to non-homogeneous gel formation [29].

**Figure 1:**
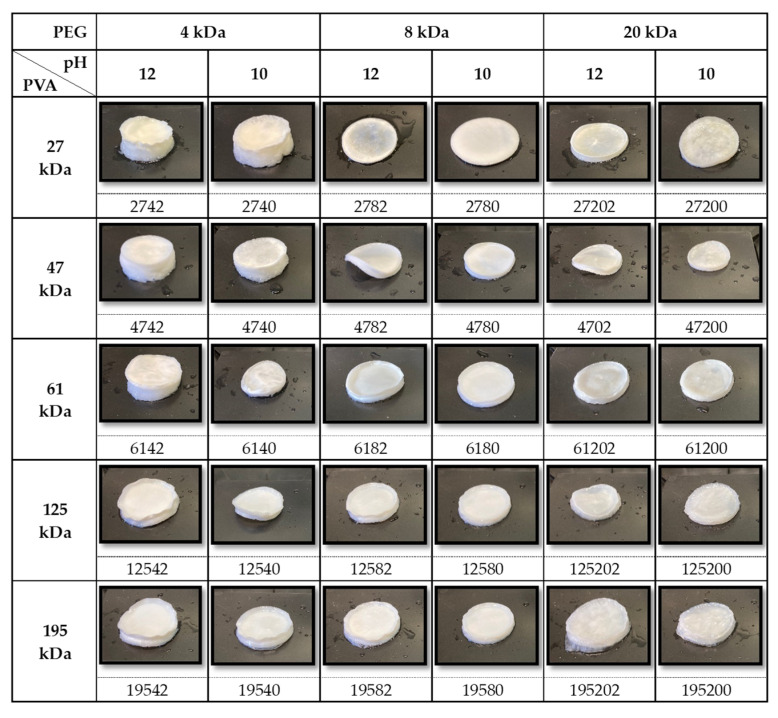
Summary of synthesized PVA-PEG theta gels. Sample labels consist of numbers that depend on PVA MW, PEG MW and pH used for the matrix preparation: XYZ is the label of each sample with X that can be 27, 47, 61, 125 or 195 kDa (PVA MW); Y that can be 4, 8 or 20 kDa (PEG MW); and Z that can be 0 or 2, for pH 10 or 12, respectively.

Darabi et al. [34] tested the preparation of PVA hydrogel in a basic environment, using a strong alkaline condition (3–6 M NaOH). Such a strong condition produced very strong hydrogels but with a too-low water content for the intended application. Huang et al. also investigated a basic environment but employed an aprotic solvent and a very high PVA concentration (i.e., 20%). We tested two different basic pHs (i.e., 10 and 12), and no significant difference was observed in terms of theta gel formation. Our results indicated that the hydrolysis degree had a major impact on theta gel formation. Specifically, 31 kDa PVA did not form any hydrogel system, whereas 205 kDa PVA gave unstable hydrogels that underwent disruption during the washing process. For both the MWs, this instability can be attributed to the lower hydrolysis degree (88% instead of 99%) with respect to the other molecular weights. The incomplete hydrolyzation of the acetate groups can hinder the proper alignment of the PVA chains, preventing them from reorganizing into a stable framework. The synthesized samples are depicted in Figure 1.

### 2.2. Swelling Behavior

**Figure 2:**
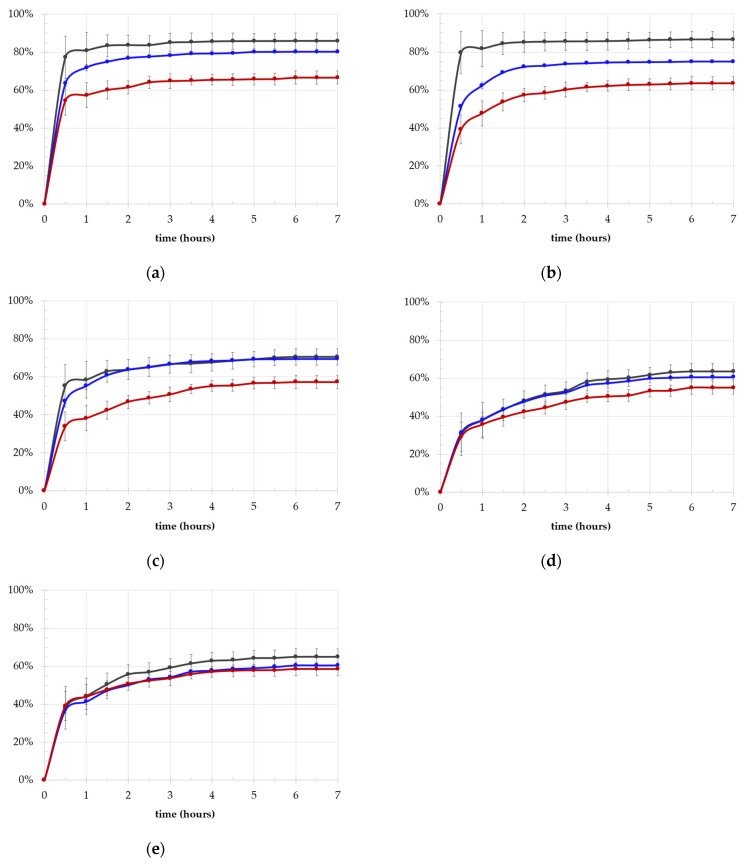
Swelling kinetic behavior in terms of SR% with PEG MW (4 kDa in dark grey, 8 kDa in blue, 20 kDa red) of PVA 27 kDa (**a**), PVA 47 kDa (**b**), PVA 61 kDa (**c**), PVA 125 kDa (**d**), PVA 195 kDa (**e**).

The results for the equilibrium swelling ratio (SR%) are presented in Figure 2a–e. All samples reached the equilibrium state within 6 h, exhibiting SR% values comparable to the water content of human cartilage, which ranges from 60% in the deepest layer to 84% in the uppermost layer [35]. Generally, the effect of increasing chain length for the two polymers was similar (*p* = 3 × 10^−7^ for PVA MW and *p* = 6 × 10^−7^ for PEG MW) leading to a decrease in SR% for all the frameworks, which shifted from 60% for HMW polymers to 85% for LMW polymer-based hydrogels. Despite this similarity, differentiating the hydrogels into low and medium PVA MW (L/M-MW, i.e., 27 kDa, 47 kDa and 61 kDa, respectively) and high MW (HMW, i.e., 125–195 kDa) revealed some differences. Specifically, L/M-MW matrices maintained the trend associated with both the polymers (*p* = 3 × 10^−6^ and *p* = 9 × 10^−5^, respectively), whereas no significant effect was observed in the H-MW samples.

**Figure 3:**
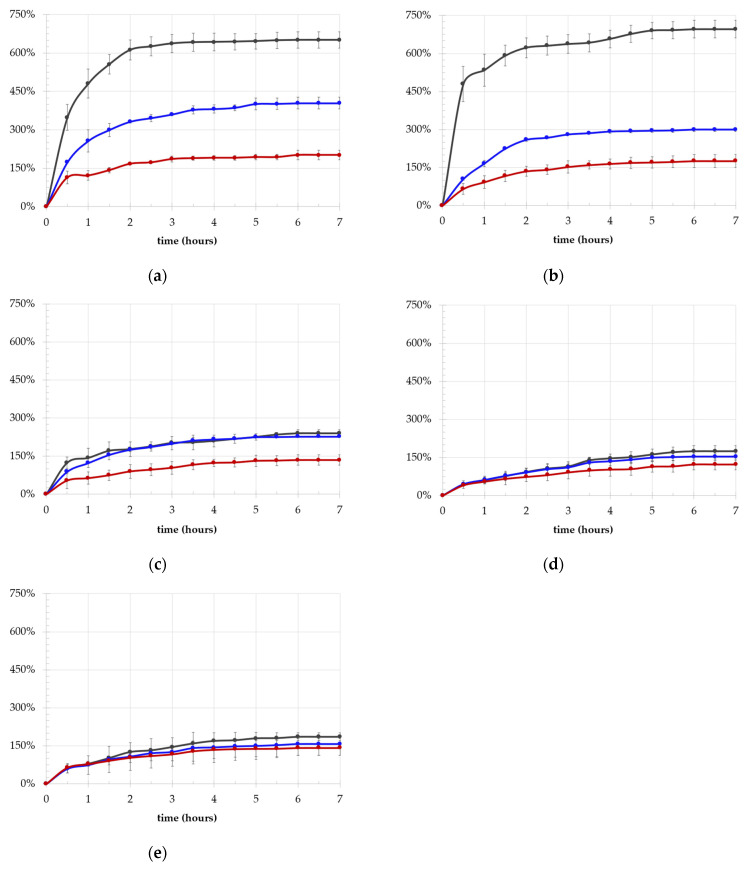
Swelling kinetic behavior in terms of UR% with PEG MW (4 kDa in dark grey, 8 kDa in blue, 20 kDa in red) of the PVA 27 kDa (**a**), PVA 47 kDa (**b**), PVA 61 kDa (**c**), PVA 125 kDa (**d**), PVA 195 kDa (**e**).

A significant difference in terms of uptake ratio (UR%) values was also found, with LMW hydrogels showing a UR of about 600% and medium- and high-MW hydrogels showing a UR value of about 200% (Figure 3).

An inverse proportionality between PVA and PEG length and UR% was also observed (*p* = 4 × 10^−6^ and *p* = 6 × 10^−6^, respectively). When differentiating the L/M-MW series from the H/UH-MW hydrogels, the dependence on PVA and PEG molecular weight remained consistent for the L and M-MW samples, although the influence of PVA length diminished somewhat (*p* = 1 × 10^−6^), while PEG length retained its significance (*p* = 5 × 10^−6^) in relation to the entire series. For the H-MW PVA series, the effect of the molecular weight of both polymers lost statistical significance, resulting in values that converged around the same average, as observed for SR%. In line with the findings of Bogoduz-Senturk et al. [24,32], who examined the role of PEG MW in water content using PEG 200 Da and PEG 6000 Da, our results indicated that in our theta gels, PEG MW significantly affected swelling capability. To further analyze the effect of PEG on the hydrophilicity of the systems, we investigated swollen and dried theta gels using a combination of TGA and DSC thermal techniques.

### 2.3. Thermal Analysis

#### 2.3.1. Hydrophilicity and Water Characterization: Swollen Theta Gels

The total water (W_H_) was determined by measuring the weight loss of swollen hydrogels with respect to the weight of the UPW fully swollen gels, in the temperature range of 30 °C to 200 °C [18]. The results presented in Table 1 indicated that, when considering the two polymers separately, PEG molecular weight significantly affected WH (*p* = 7 × 10^−3^), while PVA molecular weight did not show a significant effect. Nevertheless, the combination of the two polymers in the Two-Way ANOVA (TW-A) test showed a significant effect on the final properties of the frameworks (*p* = 8 × 10^−3^).

The total water content, as obtained from TGA analysis, is the sum of free water and bound water. The latter refers to water bound to the polymer chains (hydration water or non-freezable: W_nfH_) which facilitates the expansion of the material’s meshes and allows for the absorption of additional water (free water or freezable water: W_fH_). Freezable water can be quantified using DSC, whereas non-freezable water can be calculated by difference, as detailed in the experimental section. A higher proportion of free water corresponds to a greater swelling capacity of the material. The relative values of W_H_, W_fH_ and W_nfH_ for all the fully swollen frameworks are summarized in Table 1. W_H_ represents the mg of water per 100 mg of swollen gel of the swollen gels; W_fH_ and W_nfH_ represent the two relative contributions to the total W_H_ amount.

The varying amounts of different types of water highlighted that within the L/M-MW PVA series, both PVA (*p* = 3 × 10^−2^) and PEG (*p* = 5 × 10^−5^) MW had a significant effect, with the latter contributing more substantially. Specifically, an increase in PEG chain length resulted in a significant increase in non-freezable water, suggesting an increase in the material’s surface area. This evidence is in accordance with findings from Bogoduz-Senturk et al. which emphasized the significant role of PEG in generating and stabilizing pores, thus ensuring higher water content that remains stable even after heating treatments [24].

Additionally, a high amount of non-freezing water is crucial for the foreseen application. Since cartilage degradation is primarily caused by dehydration, the higher the hydration water of a material, the higher its resistance to dehydration, which can lead to material deterioration [36]. Based on the obtained results, the use of PEG 20 kDa as a porogen enhances non-freezing water content, thus providing the frameworks with increased resistance against dehydration due to mechanical stress.

#### 2.3.2. Thermal Stability: Dry Theta Gels

The weight loss (%) of dry samples (TG) and its first derivative (DTG) as a function of temperature are presented in Figure S1a–f. Typically, three temperature ranges are examined in polymeric materials: 30–200 °C, 200–400 °C and 400–600 °C (Table 2). The first range is associated with the evaporation of bulk and hydration water, with no other volatiles being in the sample. The second range relates to the degradation of aliphatic carbon chains, and the third one corresponds to the carbonation process, i.e., the degradation of the condensed chains of the polymeric material [37]. The R value is defined as the ratio of weight loss in the third temperature range to that in the second range. This parameter reflects the material’s degree of complexity and is directly proportional to its thermal stability and structural organization.

Interestingly, an opposite trend was observed for the dry samples. Indeed, the weight loss in the 30–200 °C range, which is associated with the hydrophilicity of the polymeric matrices, was influenced solely by PVA MW (*p* = 3 × 10^−5^), not by PEG MW. The hydrophilicity of samples decreased with an increase in PVA MW. As already observed for the swelling behavior, PVA MW maintains its influence in the PVA L/M-MW series, whereas no statistical difference is observed within the H-MW PVA series. In terms of R values, the stability of the system is slightly affected by both PVA (*p* = 0.03) and PEG (*p* = 0.02) MWs. This is consistent with PEG’s role in allowing PVA chains to come closer together, resulting in the compaction of the matrices. As observed for the weight loss (%) in the first temperature range, when dividing the frameworks by PVA molecular weight, only the L/M MW matrices showed statistically significant differences (*p* = 0.02). Furthermore, only the use of PEG 4 kDa ensured a statistically different R value within this subgroup.

#### 2.3.3. Differential Scanning Calorimetry (DSC)

**Figure 4:**
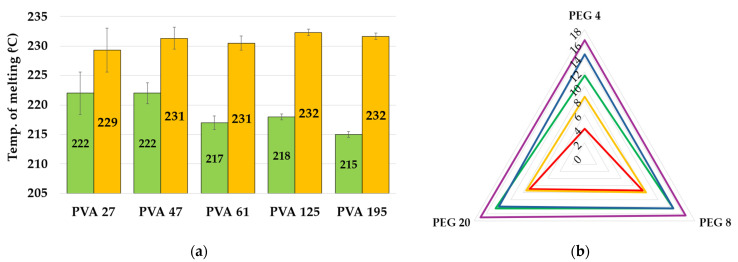
(**a**) T_m_ of the native PVA (green) and of the average of the corresponding theta gel (yellow); (**b**) ΔT_m_ (°C) between the theta gel and the corresponding PVA (red for 27 kDa, yellow for 47 kDa, green for 61 kDa, blue for 125 kDa and purple for 195 kDa).

Native PVA polymers were subjected to a heat–cool–heat cycle to erase their thermal history, allowing for the measurement of their characteristic crystallinity. On the contrary, the thermal profile of the synthesized frameworks was recorded (Figure S2a–e) using a heat-only approach, to evaluate the effect of synthesis parameters on the crystallinity of frameworks, which can be determined only during the first heating step. The enthalpy of fusion (ΔH_m_) and melting temperature (T_m_) of the polymeric matrices were obtained through DSC, and the statistical results are reported in Figure 4. Neither PVA nor PEG MW, nor their interaction, influenced the enthalpy of fusion values. ΔH_m_ values were not statistically different across the entire series of frameworks. Regarding the melting temperature, the data could not be processed as obtained, because they were not normally distributed, and no effective data transformation was achieved. For this reason, the temperatures were treated as differences (ΔT_m_) between each framework (T_m,f_) and the native PVA (T_m,n_) used for matrix preparation. The results for the two melting temperatures are shown in Figure 4a, and the ΔT_m_ values in Figure 4b.

The obtained values are primarily influenced by PVA MW (*p* = 7 × 10^−8^) and to a lesser extent by PEG (*p* = 1 × 10^−2^). An increase in ΔT_m_ is observed with increasing PVA molecular weight. When using PEG 4 kDa, ΔT_m_ differs across all samples, whereas PEG 8 kDa and 20 kDa, PVA 27 and 47 kDa (red and yellow lines, respectively), and PVA 61 kDa and 125 kDa (green and blue lines, respectively) group together with similar values. In conclusion, the frameworks exhibited an increased T_m_ (231 ± 1.0 °C versus 219 ± 3 °C), indicating enhanced melting stability in comparison with native PVA, while maintaining their crystallinity. This finding confirmed the increase in stiffness, due to the approaching of PVA chains during the phase separation, as indicated by R values obtained from TG analysis.

### 2.4. Viscoelastic Properties

The frameworks were evaluated for their viscoelastic properties, focusing on viscoelastic moduli both within and outside the linear viscoelastic region (LVR), as well as compliance (J) and recoverable compliance (J_R_). To measure these parameters, three different tests were conducted: a frequency sweep test and an amplitude sweep test for the moduli and a creep–recovery test for compliance. The viscoelastic moduli of the frameworks were measured along a range of frequencies at a constant strain. The results, presented as overall viscoelastic properties (G* = |G′ + iG″|) and the ratio of the two moduli (tan *δ* = G″/G′) [38], are summarized in Table 3. Data were extracted at 1 Hz from the results depicted in Figure S3. Given that the complex modulus is almost independent of the applied frequency, 1 Hz frequency was chosen as the midpoint of the tested frequency range.

The Two-Way ANOVA highlighted that tan *δ*, with an average value of 0.08 ± 0.02, showed no significant differences across the entire series for both PVA and PEG MWs (*p* > 0.05). Consequently, since the tan δ for all matrices is less than 0.1, all samples can be classified as predominantly elastic gels [39]. The Two-Way ANOVA test pointed out that G* is influenced by both PVA (*p* = 5 × 10^−5^) and PEG (*p* = 9 × 10^−7^) MW. Generally, the complex modulus increases with PEG MW, a trend that is particularly pronounced in the L/M MW PVA series.

**Figure 5:**
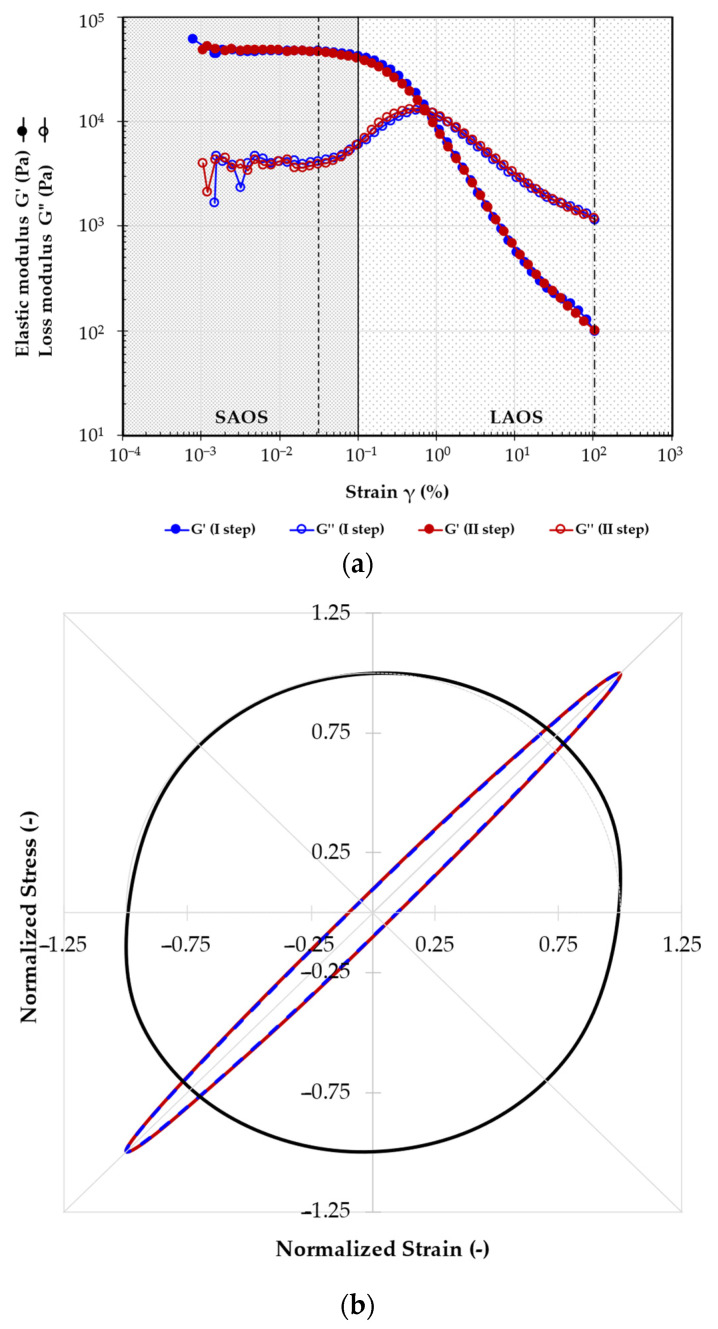
Results of the 12520 framework (**a**) Amplitude sweep test results: the full symbols refer to the increasing strain step, and the empty ones to the decreasing one. The strain at 0.05% (---, dashed line) and at 100% strain (-·-·-, point-dashed line); (**b**) the Lissajous plot of the normalized stress/strain values in the SAOS 1 (γ at 0.05% in the increasing step), SAOS 2 (γ at 0.05% in the decreasing step) and LAOS (γ at 100%). The SAOS 1 and SAOS 2 curves are the dashed red and solid blue ellipses, respectively; the LAOS curve is the black circumference-like one.

An amplitude loop test in the SAOS and LAOS regimes was conducted to assess how the frameworks respond to 100% strain and their recovery capacity. The test was performed in a continuous way, using an increasing amplitude strain ramp, followed by a decreasing ramp. Figure 5a shows the results of the amplitude sweep loop test for the 12520 framework. During this test, sine strain–stress curves were collected for each point, at 5 × 10^−2^% strain (SAOS, within the LVR) and at 100% strain (LAOS), and were then mathematically analyzed using TRIOS software v.4.1.1.33073. The Lissajous plot represents the result of this analysis. Normalized plots for SAOS in the first ramp (SAOS 1) and the second ramp (SAOS 2) and LAOS are presented as an example in Figure 3b. The plot visually shows any alterations in the mechanical properties, and the integral of the SAOS ellipsoidal curves allows for the calculation of Dissipated Energy Density (DED, in N/m^2^), which represents the deformation energy associated with the sheared volume of the sample [40].

The amplitude sweep graphs demonstrated that all the frameworks exhibit a crossover point before reaching 100% strain. Furthermore, their viscoelastic behavior significantly differs between the analyzed regions of SAOS and LAOS. From the SAOS and LAOS graph, as well as the Lissajous plots, we derived the elastic (G′) and viscous (G″) moduli, the crossover points in the first (CO 1) and second (CO 2) amplitude sweep steps, and the DED in SAOS 1 and SAOS 2. These parameters were used to discuss the mechanical behavior of the frameworks, and all values are provided in Table 4.

The amplitude sweep tests revealed a crossover behavior in all frameworks, indicating a transition from strong elastic to viscous behavior, which reverts to strong elastic behavior in the second amplitude sweep step. A One-Way ANOVA test showed no statistical differences in the same modulus, G′ and G″, between the SAOS 1 and 2 (*p* > 0.05). Therefore, the values of SAOS 1 and 2 were averaged. This analysis indicated that both PEG MW (*p* = 4 × 10^−15^ and *p* = 2 × 10^−14^, for G′ and G″, respectively) and PVA MW (*p* = 8 × 10^−14^ and *p* = 1 × 10^−9^) significantly influenced these parameters. For L/M MW PVA, an increase in PEG MW correlates with an increase in the elastic modulus. However, for PVA 195 kDa frameworks, no significant differences in moduli values were found across all PEGs. Similarly, with increasing PVA MW, the storage modulus increases. Starting from PVA 61 kDa, the elastic modulus remained constant in the M/H-MW PVA series. A similar trend was observed for the viscous modulus, though with different absolute values. Regarding the moduli under LAOS conditions, results highlighted that only PVA retained some influence, but with reduced significance, with *p*-values shifting from *p* = 8 × 10^−14^ to *p* = 3 × 10^−2^ for G′ and from *p* = 1 × 10^−9^ to *p* = 2 × 10^−3^ for G″. PEG MW lost its influence on the moduli in this strain region.

The crossover point (CO) of the frameworks is defined as the limit where the tan δ is equal to 1 (i.e., G′ = G″). This point depends on the material’s mechanical properties and on its behavior under specific stresses or strains. The reciprocal values of CO 1 and CO 2 were used for statistical analysis. A preliminary One-Way ANOVA test revealed no significant differences between CO 1 and CO 2 values for all frameworks, leading to the merging of the two variables. All data are summarized in Table 4. It is possible to affirm that for 1/CO, the overall behavior for all frameworks mirrors that of G′ under SAOS conditions. For this parameter, as for the G′ value, PEG molecular weight emerged as the most influential variable, with *p* = 4 × 10^−11^, compared to *p* = 7 × 10^−4^ for PVA MW. DED analysis allowed us to determine if the material sustained damage or lost mechanical properties after the loop procedures. The data were analyzed as the ratio of DED 2 normalized to the corresponding DED 1 (DED 2/DED 1). A Two-Way ANOVA test was conducted to assess the influence of PVA and PEG on this parameter. Only PVA MW showed a slight effect on the DED ratio (*p* = 0.02), while PEG MW had no effect. From Table 4, it is evident that for PEG 4 kDa, the DED ratio values remained relatively constant across the entire PVA series. For PEG 8 kDa, a marked difference is observed between L-MW and M/H-MW PVAs, with the latter showing a higher DED ratio value. For PEG 20 kDa, results indicated that all PVAs exhibited similar DED ratio values, except for the 27 kDa PVA, which maintained a nearly constant DED ratio across the PEG series.

This parameter was analyzed to evaluate the material’s ability to dissipate impact energy density. This property permits a material to resist impact which could induce microcracking and affect the material’s performance. Specifically, the effect of 1.0 N/m^2^ energy density impact was examined, as it poses a significant risk of microcracking within the collagen network, potentially leading to degenerative conditions such as post-traumatic osteoarthritis, as noted in the study by Khaleem et al. [41]. Kaleem et al. performed 108 low-energy impact tests on healthy cartilage specimens and found that an impact energy density of 1.0 N/m^2^ corresponded to a ∼20% probability of microcracking [41].

Based on this, the averaged values for the 6120 and 12520 frameworks, identified as the most promising materials for the intended application, showed a dissipation energy density of 1N/m^2^ from a strain value of 0.5%, with DEDs of 1.3 N/m^2^ ± 0.0 N/m^2^ and 1.2 N/m^2^ ± 0.0 N/m^2^, respectively.

The creep–recovery test was conducted to evaluate the behavior of the frameworks, in terms of compliance (J), when subjected to a constant stress within the LVR, as well as their subsequent recoverable compliance (JR). The recovered compliance (J_RE_) was calculated as the percentage of the recoverable compliance relative to the corresponding compliance. The results at a steady state are reported in Table 5.

**Figure 6:**
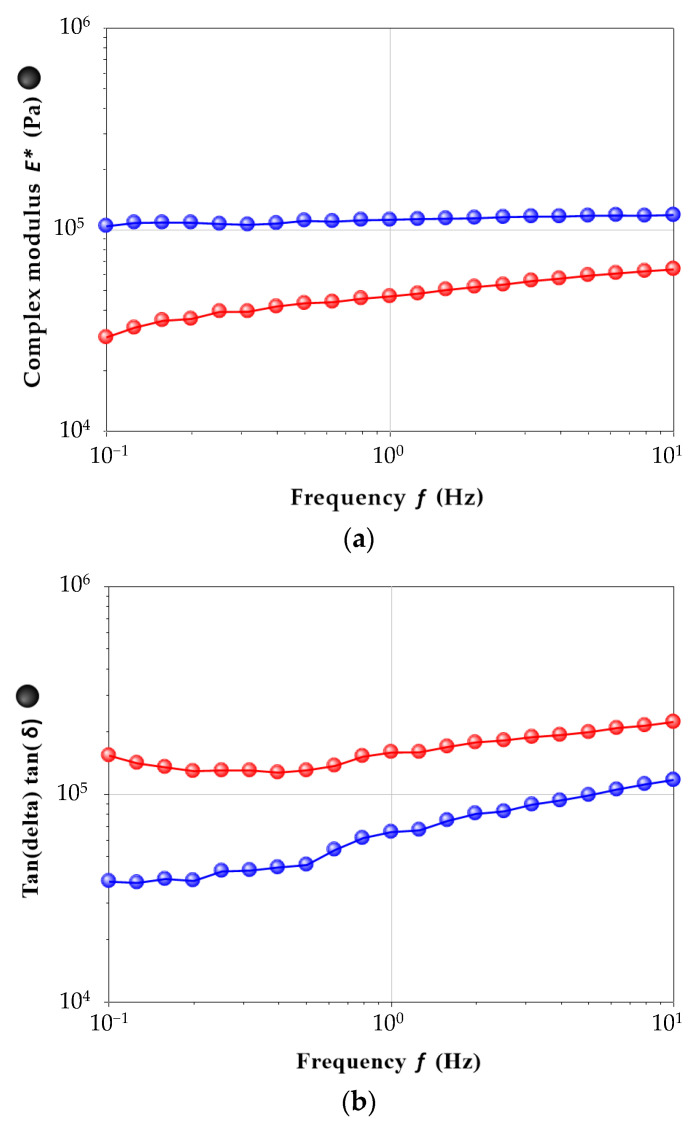
(**a**) E* modulus and (**b**) tan δ of PVA 6120 (blue) and PVA 12520 (red) as a function of increasing frequency (equilibration time: 120 s).

The TW-A was conducted on the reciprocal of the compliance (1/J), revealing significant effects from both PVA and PEG MW, with *p*-values of 3 × 10^−3^ and 4 × 10^−2^, respectively. The parameter exhibited two distinct behaviors across the PVA series. Generally, the L/M-MW group showed a decrease in 1/J with increasing PEG MW, while the H-MW PVA series maintained relatively constant compliance values across all PEGs. In terms of the reciprocal of the recoverable compliance (1/J_R_), PEG had a greater influence on this parameter than PVA, with *p*-values of 4 × 10^−3^ and 2 × 10^−2^, respectively. Instead of focusing solely on recoverable compliance, it is more informative to analyze the recovered compliance, which is the recoverable compliance relative to the corresponding compliance (J_RE_ = J_R_/J). Our materials exhibited an average recovered compliance of 56 ± 22%, close to the findings from Choi et al., who measured an average value of 57% for PVA-PEG theta gels [42], and Bogoduz-Senturk et al., who found values ranging from 61% to 69% [24,32]. Statistical analyses indicated that the porogen molecular weight plays a crucial role in these hydrogels’ characteristics, consistent with previously published studies highlighting the significant effect of PEG compared to PVA [24,32]. However, samples made with 61 kDa and 125 kDa PVA demonstrated the highest percentages of recoverable compliance, suggesting they are the most suitable materials for the foreseen application. Additionally, as indicated by shear modulus measurements (Table 3), only the 6120 and 12520 frameworks appear appropriate for cartilage tissue engineering since their equilibrium shear modulus falls within the range of native articular cartilage (50–250 kPa) [43]. Consequently, the 61 kDa and 125 kDa PVA theta gels, prepared using PEG 20 kDa as porogen, were further analyzed in terms of compression moduli and coefficient of friction. In Figure 6, the trends of E* and tan δ as a function of increasing frequency are depicted. To minimize the noise associated with the viscous component (E″), which is more sensitive to frequency during compression measurements, various equilibration times were tested, measuring the behavior after 60 s, 120 s, 180 s, 240 s and 300 s. Adequate equilibration was achieved after 120 s. No differences were observed when increasing the equilibration time to 180 s, 240 s and 300 s. Sample 12520 demonstrated an optimal mechanical response under compression across the entire analyzed frequency range with a mean value of 0.12 MPa.

**Figure 7:**
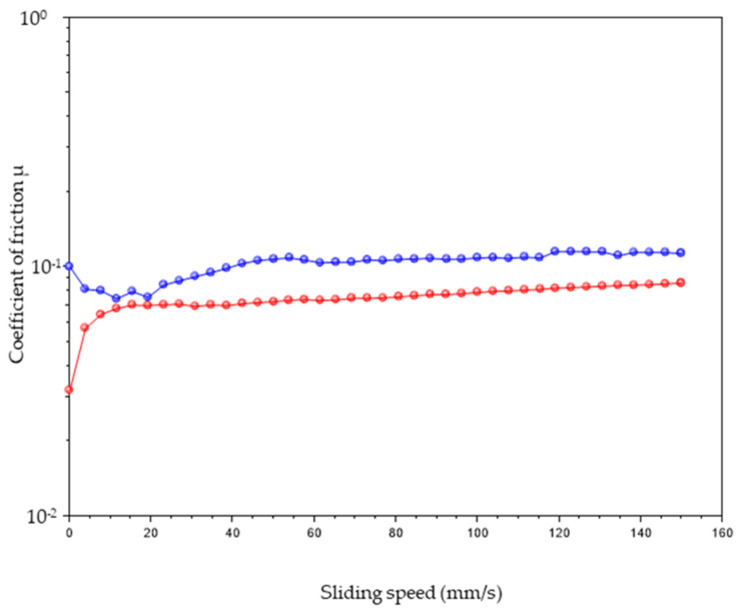
Coefficient of friction (COF) of the samples 6120 (blue) and 12520 (red).

One of the most important properties of articular cartilage is its low friction [44,45]. To measure the COF for cartilage substitute hydrogels, various constant loads have been studied [44,45,46,47,48]. However, most of the studies report COF values obtained using constant loads ranging from 2.0 N to 10 N. This range of values is typically used to generate pressures close to the normal loads experienced by articular cartilage (0.1–2.0 MPa) [49,50]. Based on this, we selected a mean value of 4.5 N to facilitate comparisons with COF values reported for similar materials. The COF of the selected frameworks, measured at a constant load of 4.5 N, is depicted in Figure 7.

The coefficient of friction of articular cartilage has been reported to be around 10^−2^, at a sliding velocity of 10 mm/s under a constant load of 4.5 N [46,47]. However, significant variability in COF values has been observed, depending on sample shape. Indeed, Krishnan et al. [46] measured the friction coefficient in bovine articular cartilage subjected to a 4.5 N constant load, finding a COF that ranged from a minimum of 0.010 ± 0.007 to a maximum of 0.243 ± 0.044. They fitted the experimental data to a biphasic boundary lubrication model, resulting in an equilibrium friction coefficient of 0.284 ± 0.044. Mahmood et al. [47] measured the coefficient of friction of articular cartilage under a 5 N load, reporting an average value of approximately 0.36. Ye et al. [30] assessed COF values for PVA-PEG hybrid gels at various loads (i.e., 3 N, 5 N, 8 N and 1 0N) and found a low dependence of the COF on increasing load, with values varying from 0.20 to 0.12 at 5.0 N.

Between the two selected frameworks, sample 12520 exhibited a more favorable trend, maintaining a COF below 0.10 for all tested sliding speeds (mm/s) [48].

## 3. Materials and Methods

### 3.1. Materials

PVA (27 kDa, 47 kDa, 6 1kDa, 125 kDa, 195 kDa: 99% hydrolysis degree; 31 kDa, 205 kDa: 88% hydrolysis degree), PEG (4 kDa, 8 kDa and 20 kDa), NaOH and all solvents and chemicals were purchased from Merck and used without further purifications.

### 3.2. PVA Framework Synthesis

Polymeric frameworks were synthesized by combining a 10% *w*/*w* PVA solution with a 10% *w*/*w* PEG solution in ultrapure water (UPW). Both solutions were basified with 1 M NaOH to achieve the desired pH (pH = 10 or pH = 12) After a resting period of 30 min, the two solutions were mixed vigorously and allowed to rest for 24 h to facilitate PVA re-crystallization. Subsequently, the solution was removed, and the samples were freeze-dried. The dry samples were then washed with UPW to remove PEG and residual NaOH. A total of 42 frameworks were obtained by varying PVA and PEG molecular weights and pH conditions.

### 3.3. Swelling Behavior

The swelling behavior of all frameworks was assessed in a physiological solution at 37 °C. Approximately, 20 mg of each sample in its dry state was immersed in an excess of 154 mM NaCl, and weight changes were monitored every 30 min for the first 8 h, and subsequently after 24, 48 and 72 h. The swelling behavior was expressed as the swelling ratio (SR%) and uptake ratio (UR%) [9,12]. The SR% is defined in Equation (1).

(1) $$\mathrm{SR}\%=\frac{W_{s}-W_{d}}{W_{s}}\;\cdot 100$$

The UR% is defined in Equation (2).

(2) $$\mathrm{UR}\%=\frac{W_{s}-W_{d}}{W_{d}}\;\cdot 100$$

where W_s_ and W_d_ correspond to the weight of the swollen and dried matrix, respectively.

### 3.4. Thermal Analysis

#### 3.4.1. Thermogravimetric Analysis (TGA)

TGA was employed to quantify the total water content and the thermal stability of the frameworks. The analyses were conducted using an SDT-Q600 (TA Instruments, Leatherhead, UK) with Thermal Advantage Release 5.5.22 for instrument control and TA Instruments Universal Analysis 2000 v. 4.5.4. for data analysis. The total water content (WH) of each sample was assessed by heating 10 mg of the fully swollen gels in UPW from 30 °C to 300 °C at 10 °C/min.

Thermal stability was quantified by heating 10 mg of the dry-state frameworks from 30 °C to 600 °C at 10 °C/min. Both tests were performed in an inert atmosphere (N_2_, 100 mL/min) [51].

#### 3.4.2. Differential Scanning Calorimetry (DSC)

DSC analyses were conducted on both swollen and dried hydrogels using a DSC Q1000 (TA Instruments). The instrument was controlled using Thermal Advantage Release 5.5.22, and data were analyzed with TA Instruments Universal Analysis 2000 v. 4.5.4. For the analysis, 5 mg of each ultra-pure water (UPW) swollen gel was sealed in an aluminum hermetic anodized pan. The thermal program included cooling the sample to −40 °C (0.2 °C/min.), holding for 5 min at −40 °C, and then heating from −40 °C to 40 °C at 0.2 °C/min. All steps were conducted under a N_2_ flow of 50 mL/min. The total water content is expressed in Equation (3).

(3) $$W_{H}=W_{\mathrm{fH}}-W_{\mathrm{nfH}}$$

where W_H_, W_fH_ and W_nfH_ are the weight of total water in the UPW full swollen sample, the weight of freezable and the weight of non-freezable water, respectively. The freezable water is determined by integrating the endothermic peak corresponding to the melting of the frozen water in the hydrogel sample (ΔH_m_) and quantifying the latent heat of melting of pure free water (ΔH). The ratio ΔH_m_/ΔH provides the weight of freezable water per gram of full swollen hydrogel (W_s_), equation (4) [51].

(4) $$\frac{W_{\mathrm{fH}}}{W_{s}}=\frac{{∆H}_{m}}{∆H}$$

By applying Equation (4), it is possible to calculate the W_fH_, and from Equation (3), the W_nfH_.

Then, 1–5 mg of the dry-state framework was sealed in a hermetic anodized aluminum pan and heated from 10 °C to 250 °C at a heating rate of 10 °C/min under N_2_ flux of 50 mL/min to obtain the melting behavior.

### 3.5. Viscoelastic Properties

The mechanical behavior of the frameworks was analyzed through rheological tests, focusing on Small Amplitude Oscillation Shear (SAOS) and Large Amplitude Oscillation Shear (LAOS) regimes. For all the analyses, the samples were analyzed in the full swollen state in 154 mM NaCl at 37 °C. Tests were performed using a Discovery Hybrid Rheometer-2 (DHR-2) (TA Instruments) equipped with a stainless-steel plate–plate geometry (diameter 40 mm) and a Peltier plate environmental system for temperature control. Instrument control and data analysis were carried out using TA Instruments software TRIOS v.4.1.1.33073.

#### 3.5.1. Frequency Sweep Test (SAOS)

Samples underwent a strain sweep test with strains ranging from 0.01% to 5% at three frequencies (0.1 Hz, 1 Hz and 10 Hz) to determine the linear viscoelastic region (LVR). The frequency sweep test was then performed from 0.1 Hz to 10 Hz at a constant strain of 0.05% (equilibration time 60 s) as deduced by the strain sweep test.

Selected samples (6120 and 12520) were tested also in compression mode from 0.1 Hz to 15 Hz at a constant strain of 0.05%, as deduced by the strain sweep test, and varying equilibration times (i.e., 60 s, 120 s, 180 s, 240 s and 300 s).

#### 3.5.2. Amplitude Sweep Test (SAOS and LAOS)

A strain sweep test was performed at a constant frequency of 1 Hz, applying two consecutive strain ramps: the first one from 0.001% to 100%, followed by a return ramp from 100% to 0.001%.

#### 3.5.3. Creep–Recovery Test

The creep step was performed at a constant stress (σ) of 50 Pa (selected based on preliminary tests indicating it was within the LVR) for 90 s. Following this, the stress was removed (0 Pa), and the material’s recovery response was recorded for an additional 180 s. The durations of the two steps were optimized based on preliminary tests that indicated a steady state was achieved within these timeframes.

#### 3.5.4. Tribological Properties

**Figure 8:**
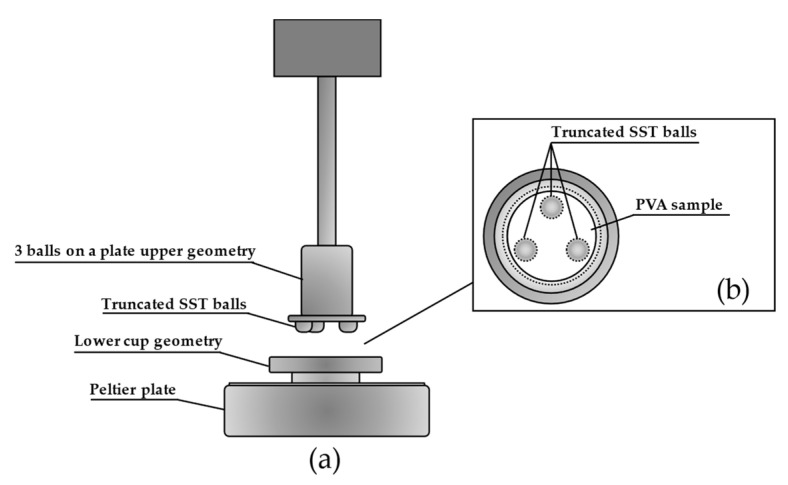
(**a**) Tribological set-up for the COF analysis; (**b**) upper view of the tribological geometry with the upper one drawn with a short dash line and the PVA sample in white.

Tribological tests were performed on selected swollen matrices in 154 mM NaCl at 37 °C using the DHR-2, equipped with an upper geometry of three 5/16″ stainless steel truncated balls (Figure 8). The test was conducted as a flow sweep, ranging from 0.01 rad/s to 10 rad/s, under a load force of 4.5 N, allowing the determination of the coefficient of friction (COF) [46], represented by *μ*. The COF is defined as the ratio of the friction force (F_R_) to the loading force (F_L_).

### 3.6. Statistical Analysis

For the statistical analysis, three replicates were utilized for each measurement. The effect of pH on the properties of the materials was assessed using a One-Way ANOVA test. If no significant differences were found (*p* > 0.05) among the results for the same sample, the data were averaged, effectively increasing the number of replicates from n = 3 to n = 6. The influence of molecular weights of both PVA and PEG on the analyzed properties was examined using a Two-Way ANOVA test. The results were graphed following this statistical analysis, and any transformations necessary to normalize the data were applied.

## 4. Conclusions

In this study, we explored the possibility of obtaining PVA-based theta gels for use in treating diseased cartilage. The theta gels were prepared using mild conditions consisting of an aqueous environment and room temperature while working at basic pH (i.e., 10 and 12). Our findings indicated that the degree of hydrolysis of native PVA significantly influenced the formation of stable 3D matrices. Specifically, a low hydrolysis degree (87%) inhibited the PVA chains approaching, preventing the establishment of stable frameworks. We also investigated the effects of PVA and PEG molecular weights, as well as pH values, on the water interaction, thermal properties and rheological behavior of the theta gels using a statistical approach. The statistical analysis highlighted that pH values had no significant impact on swelling, thermal and rheological behavior. The molecular weight of PEG primarily influenced the hydrophilic properties of the materials in their swollen state, whereas the thermal properties measured on dried samples were predominantly affected by PVA MW. Both polymer molecular weights impacted the shear and compression mechanical behavior of the gels. The sample prepared with PVA 125 kDa and PEG 20 kDa as a porogen emerged as the most suitable for cartilage disease treatment, exhibiting an equilibrium shear modulus in the range of 50–250 kPa, similar to that of native articular cartilage. Additionally, it demonstrated an optimal mechanical response under compression across the entire frequency range analyzed, with a mean value of 0.12 MPa. The coefficient of friction (COF) under a 4.5 N load remained below 0.10 for all tested sliding speeds.

In conclusion, the developed procedure allowed for the preparation of PVA theta gels with suitable properties for the intended application without the use of an aprotic solvent, high temperatures, or strong alkaline conditions.

## Figures and Tables

**Table 1:** Different types of water in the UPW full swollen samples. Sample labels (Sample ID) consist of numbers that depend on PVA MW and PEG MW. The reference to pH used for the matrix preparation has been removed since the pH has no effect. XY is the label of each sample, with X that can be 27, 47, 61, 125 or 195 kDa (PVA MW) and Y that can be 4, 8 or 20 kDa (PEG MW).

Sample ID	W_H_%	W_fH_% *	W_nfH_%
274	89% ± 3%	81% ± 6%	19% ± 3%
278	84% ± 1%	69% ± 5%	31% ± 6%
2720	75% ± 5%	57% ± 6%	43% ± 6%
474	94% ± 5%	80% ± 3%	20% ± 1%
478	79% ± 3%	62% ± 17%	38% ± 4%
4720	67% ± 7%	57% ± 4%	43% ± 5%
614	83% ± 2%	71% ± 17%	29% ± 3%
618	77% ± 3%	62% ± 2%	38% ± 4%
6120	67% ± 3%	46% ± 6%	54% ± 1%
1254	75% ± 2%	63% ± 9%	37% ± 1%
1258	74% ± 1%	56% ± 3%	44% ± 1%
12520	72% ± 6%	51% ± 10%	49% ± 1%
1954	74% ± 3%	58% ± 7%	42% ± 1%
1958	70% ± 4%	49% ± 4%	51% ± 4%
19520	87% ± 17%	50% ± 7%	50% ± 1%

* Data are not statistically different, *p* > 0.05.

**Table 2:** Weight losses in the characteristic temperature ranges for polymers.

Sample ID	Weight Loss (%)			R (-)
	30–200 °C	200–400 °C	400–600 °C	
274	6.0% ± 0.2%	79.3% ± 0.4%	15.4% ± 1.3%	0.21 ± 0.02
278	5.6% ± 0.3%	65.6% ± 5.6%	24.1% ± 7.3%	0.37 ± 0.14
2720	5.9% ± 0.2%	68.3% ± 1.5%	22.1% ± 2.5%	0.32 ± 0.04
474	5.3% ± 0.0%	72.9% ± 0.3%	17.7% ± 0.3%	0.24 ± 0.00
478	5.1% ± 0.9%	66.2% ± 0.3%	24.4% ± 0.4%	0.37 ± 0.00
4720	5.3% ± 0.7%	67.4% ± 2.3%	23.9% ± 2.4%	0.36 ± 0.05
614	5.1% ± 0.4%	67.9% ± 1.2%	22.1% ± 0.0%	0.32 ± 0.01
618	4.4% ± 0.5%	65.5% ± 4.1%	26.6% ± 4.8%	0.41 ± 0.10
6120	4.4% ± 0.3%	67.0% ± 0.4%	24.7% ± 1.5%	0.37 ± 0.02
1254	4.2% ± 0.1%	66.1% ± 3.1%	26.0% ± 2.0%	0.40 ± 0.04
1258	4.5% ± 0.2%	67.3% ± 1.0%	25.4% ± 0.8%	0.38 ± 0.01
12520	3.9% ± 0.2%	65.8% ± 4.7%	25.3% ± 0.8%	0.39 ± 0.04
1954	4.2% ± 0.5%	66.4% ± 1.1%	27.0% ± 1.0%	0.40 ± 0.03
1958	4.6% ± 0.5%	66.0% ± 0.6%	27.0% ± 1.0%	0.41 ± 0.02
19520	4.1% ± 0.3%	67.8% ± 0.2%	25.2% ± 0.7%	0.37 ± 0.01

**Table 3:** Frequency sweep test results in terms of G* and tan *δ* at 1 Hz.

Sample ID	G* (Pa)	tan δ (-)
274	3243 ± 3508	6.7 × 10^−2^ ± 4.4 × 10^−3^
278	16,452 ± 5589	9.8 × 10^−2^ ± 4.4 × 10^−2^
2720	29,869 ± 3962	7.6 × 10^−2^ ± 1.7 × 10^−2^
474	5383 ± 482	6.9 × 10^−2^ ± 7.0 × 10^−3^
478	15,677 ± 4793	6.2 × 10^−2^ ± 1.5 × 10^−2^
4720	21,588 ± 2915	7.8 × 10^−2^ ± 1.5 × 10^−2^
614	7227 ± 3899	5.7 × 10^−2^ ± 1.4 × 10^−3^
618	22,221 ± 10,124	6.5 × 10^−2^ ± 1.1 × 10^−2^
6120	53,202 ± 3817	8.8 × 10^−2^ ± 1.2 × 10^−2^
1254	23,079 ± 7747	4.8 × 10^−2^ ± 4.4 × 10^−3^
1258	27,394 ± 5473	8.4 × 10^−2^ ± 5.3 × 10^−3^
12520	44,305 ± 1932	9.5 × 10^−2^ ± 6.1 × 10^−3^
1954	35,314 ± 12,235	8.1 × 10^−2^ ± 2.6 × 10^−3^
1958	27,432 ± 2341	8.6 × 10^−2^ ± 2.0 × 10^−2^
19520	36,906 ± 3142	8.5 × 10^−2^ ± 1.7 × 10^−2^

**Table 4:** Results of the amplitude sweep analyses in LAOS and SAOS 1 and SAOS 2 for the increasing and decreasing strain, respectively.

Sample ID	G′ (Pa)			G″ (Pa)			Cross-Over Strain (%)		Dissipated Energy Density (N/m^2^)	
	SAOS 1 ^I^	LAOS ^II^	SAOS 2 ^I^	SAOS 1 ^I^	LAOS ^II^	SAOS 2 ^I^	CO 1	CO 2	DED 1 ^I^	DED 2 ^I^
274	3237 ± 3501	47 ± 0	2843± 2776	209 ± 220	254 ± 180	220 ± 216	8.5 ± 6.6	5.9 ± 5.2	1.9 × 10^−4^ ± 1.3 × 10^−4^	1.6 × 10^−4^ ± 1.4 × 10^−4^
278	16,353 ± 5489	36 ± 26	10,091 ± 1878	1729 ± 1264	419 ± 137	865 ± 450	2.7 ± 1.9	1.8 ± 1.5	1.4 × 10^−3^ ± 9.8 × 10^−4^	6.7 × 10^−4^ ± 3.7 × 10^−4^
2720	29,778 ± 3912	21 ± 7	20,024 ± 13,105	2303 ± 792	483 ± 180	1679 ± 1384	1 ± 0.0	0.8 ± 0.3	1.1 × 10^−1^ ± 1.6 × 10^−1^	1.0 × 10^−1^ ± 1.4 × 10^−1^
474	5370 ± 483	138 ± 19	3845 ± 1408	367 ± 4	559 ± 162	245 ± 135	7.7 ± 0.3	8.2 ± 0.9	2.4 × 10^−4^ ± 6.9 × 10^−5^	1.9 × 10^−4^ ± 1.0 × 10^−4^
478	15,643 ± 4769	53 ± 30	10,390 ± 1906	1005 ± 536	607 ± 146	511 ± 226	3.3 ± 1.5	2 ± 1.2	7.9 × 10^−4^ ± 4.3 × 10^−4^	4.0 × 10^−4^ ± 1.8 × 10^−4^
4720	21,524 ± 2930	54 ± 36	19,302 ± 4795	1651 ± 87	623 ± 145	1701 ± 45	1.7 ± 0.6	1.2 ± 0.4	1.3 × 10^−3^ ± 7.8 × 10^−5^	1.3 × 10^−3^ ± 2.1 × 10^−5^
614	7215 ± 3892	135 ± 18	7343 ± 3758	417 ± 234	692 ± 313	433 ± 306	7 ± 2.1	6.9 ± 2.3	3.4 × 10^−4^ ± 2.2 × 10^−4^	3.4 × 10^−4^ ± 2.3 × 10^−4^
618	22,177 ± 10,118	173 ± 162	23,496 ± 14,249	1392 ± 415	1289 ± 880	1353 ± 894	1.9 ± 0.1	1.5 ± 0.1	6.6 × 10^−2^ ± 9.2 × 10^−2^	7.7 × 10^−2^ ± 1.1 × 10^−1^
6120	52,991 ± 3745	103 ± 10	47,588 ± 1881	4703 ± 979	1101 ± 18	5295 ± 1953	0.9 ± 0.0	0.6 ± 0.0	2.2 × 10^−1^ ± 3.0 × 10^−1^	2.5 × 10^−1^ ± 3.5 × 10^−1^
1254	23,051 ± 7733	141 ± 20	21,557 ± 6610	1133 ± 476	1100 ± 37	1025 ± 451	1.9 ± 0.4	1.5 ± 0.5	9.2 × 10^−4^ ± 4.0 × 10^−4^	7.9 × 10^−4^ ± 3.3 × 10^−4^
1258	27,300 ± 5465	230 ± 163	27,030 ± 3651	2270 ± 313	1431 ± 547	2128 ± 489	1.9 ± 1.1	1.6 ± 0.7	1.8 × 10^−3^ ± 2.8 × 10^−4^	1.6 × 10^−3^ ± 3.7 × 10^−4^
12520	44,107 ± 1898	105 ± 7	43,424 ± 1171	4174 ± 450	1135 ± 32	4087 ± 242	0.8 ± 0.0	0.7 ± 0.0	3.3 × 10^−3^ ± 2.8 × 10^−4^	3.1 × 10^−3^ ± 1.5 × 10^−4^
1954	35,199 ± 12,188	122 ± 18	35,055 ± 11,615	2853 ± 1074	960 ± 94	2408 ± 837	1.3 ± 0.6	1 ± 0.5	2.3 × 10^−3^ ± 8.6 × 10^−4^	1.8 × 10^−3^ ± 5.7 × 10^−4^
1958	27,326 ± 2285	122 ± 15	27,949 ± 2142	2371 ± 753	1038 ± 88	2388 ± 742	1.7 ± 0.6	1.5 ± 0.2	1.9 × 10^−3^ ± 8.6 × 10^−4^	1.8 × 10^−3^ ± 5.4 × 10^−4^
19520	36,773 ± 3183	47 ± 17	34,876 ± 3244	3103 ± 350	697 ± 207	3738 ± 1209	1 ± 0.1	0.9 ± 0.1	1.4 × 10^−1^ ± 1.9 × 10^−1^	1.7 × 10^−1^ ± 2.4 × 10^−1^

The strain conditions correspond to: ^I^ γ = 0.05%, ^II^ γ = 1

**Table 5:** Creep–recovery test results at a steady state.

Sample ID	Compliance ^I^ (J, Pa)	Recoverable Compliance ^II^ (J_R_, Pa)	Recovered Compliance (J_RE_, Pa)
274	1156 ± 776	3118 ± 1242	35% ± 11%
278	3328 ± 3599	6392 ± 81	52% ± 56%
2720	5813 ± 1410	10,757 ± 4058	56% ± 8%
474	1548 ± 1911	2952 ± 1421	42% ± 45%
478	7008 ± 4834	12,445 ± 5356	53% ± 16%
4720	5542 ± 712	14,688 ± 4769	39% ± 8%
614	4141 ± 2678	5411 ± 2954	74% ± 9%
618	6400 ± 1890	10,348 ± 4463	64% ± 9%
6120	11,088 ± 3557	25,136 ± 2401	44% ± 10%
1254	13,882 ± 4802	16,212 ± 5323	85% ± 2%
1258	11,827 ± 1086	15,358 ± 2306	77% ± 5%
12520	10,016 ± 5773	15,747 ± 8329	63% ± 4%
1954	6369 ± 2264	14,059 ± 9213	51% ± 17%
1958	4078 ± 913	14,249 ± 10,622	43% ± 38%
19520	13,789 ± 4009	23,045 ± 3482	59% ± 8%

The step conditions correspond to: ^I^ σ = 50 Pa at 90 s, ^II^ σ = 0 Pa at 180 s.

## Data Availability

Data will be made available on request.

## Supplementary Materials

The following supporting information can be downloaded at: https://www.mdpi.com/article/10.3390/ijms252212039/s1.
