# Supplementary material for: Effect of Mild Conditions on PVA-Based Theta Gel Preparation: Thermal and Rheological Characterization
Source: Int J Mol Sci. 2024 Nov 9;25(22):12039. doi: 10.3390/ijms252212039 (PMC11593468; doi:10.3390/ijms252212039)
Supplement: Supplementary file 1 [file ijms-25-12039-s001.zip › ijms-3242745-supplementary.pdf]

Supporting Materials

Effect of Mild Conditions on PVA-Based Theta Gel Preparation: Thermal and Rheological Characterization

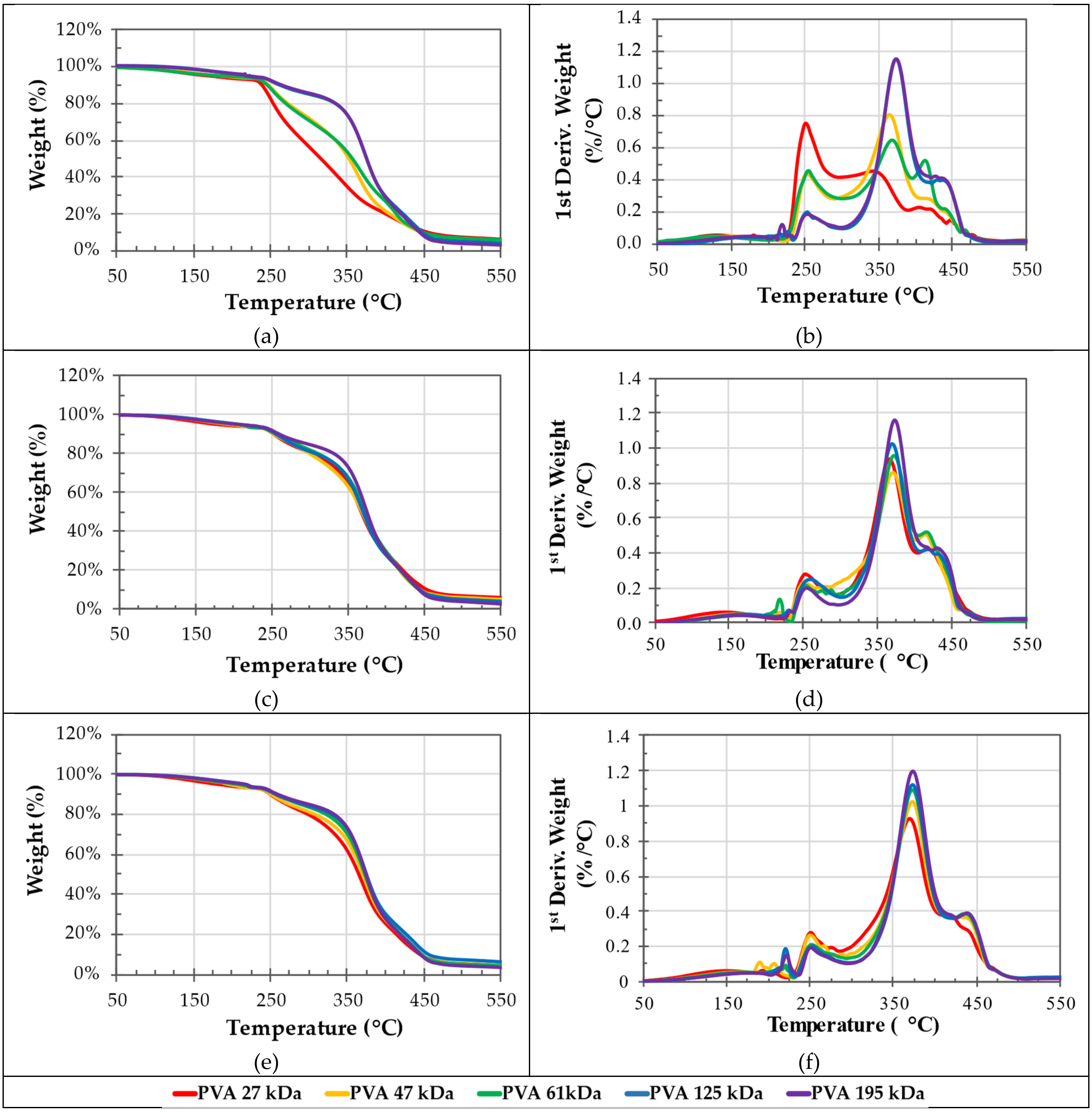

Figure S1: Averaged TGA (a, c, e) and SDT (b, d, f), grouped by PEG: 4 kDa in the first row, 8 kDa in the second one, 20 kDa in the last one.

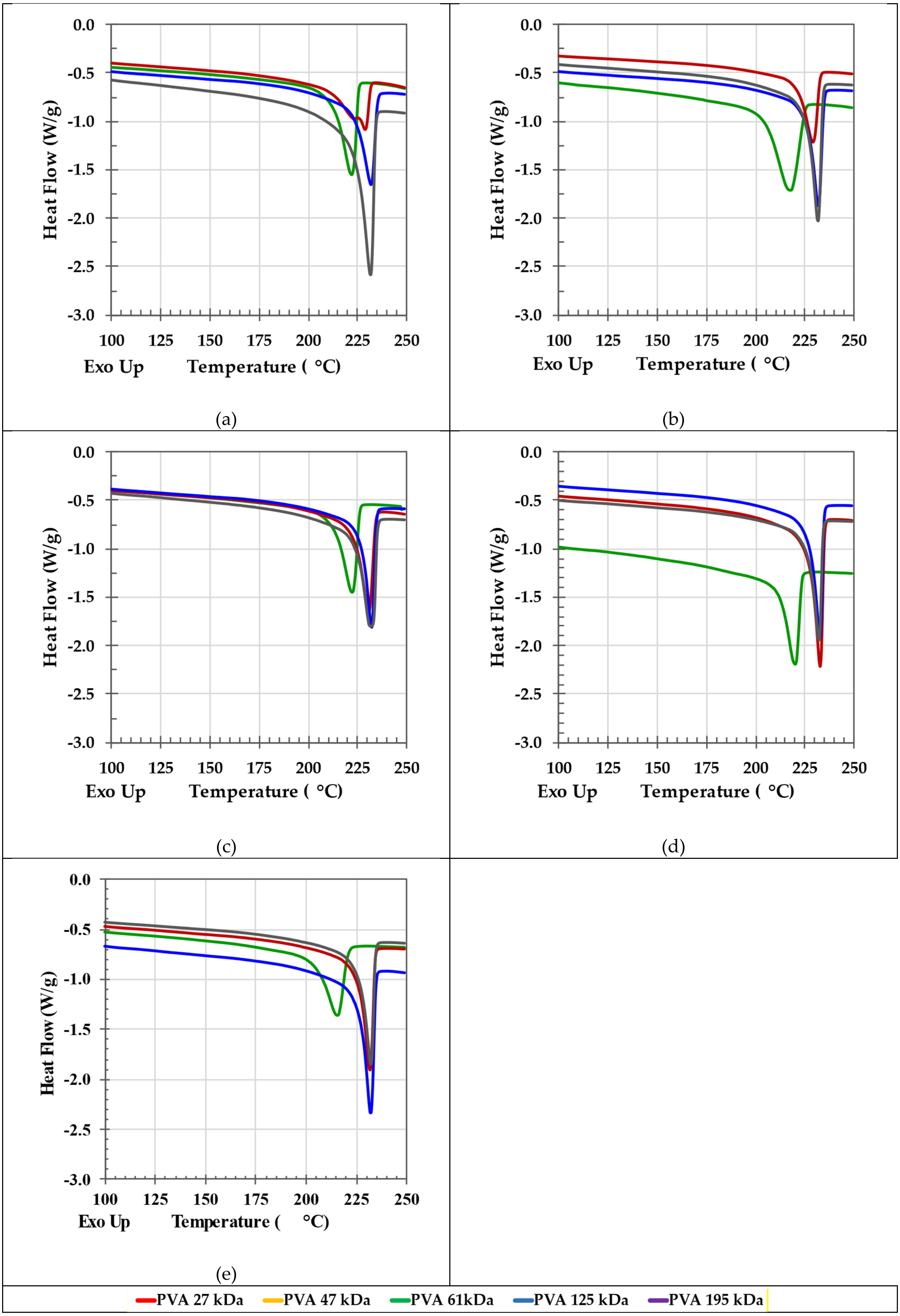
0.0
-0.5
-1.0
-1.5
-2.0
-2.5
-3.0
Heat Flow (W/g)
100
125
150
175
200
225
250
Exo Up
Temperature (°C)
(a)
0.0
-0.5
-1.0
-1.5
-2.0
-2.5
-3.0
Heat Flow (W/g)
100
125
150
175
200
225
250
Exo Up
Temperature (°C)
(b)
0.0
-0.5
-1.0
-1.5
-2.0
-2.5
-3.0
Heat Flow (W/g)
100
125
150
175
200
225
250
Exo Up
Temperature (°C)
(c)
0.0
-0.5
-1.0
-1.5
-2.0
-2.5
-3.0
Heat Flow (W/g)
100
125
150
175
200
225
250
Exo Up
Temperature (°C)
(d)
0.0
-0.5
-1.0
-1.5
-2.0
-2.5
-3.0
Heat Flow (W/g)
100
125
150
175
200
225
250
Exo Up
Temperature (°C)
(e)
PVA 27 kDa
PVA 47 kDa
PVA 61kDa
PVA 125 kDa
PVA 195 kDa

Figure S2: The averaged DSC melting thermograms of the frameworks, grouped by PVA MW, with PEG MW (4 kDa in red, 8 kDa in blue, 20 kDa in dark grey, native PVA in green) of the PVA 27 kDa (a), PVA 47 kDa (b), PVA 61 kDa (c), PVA 125 kDa (d), PVA 195 kDa (e).

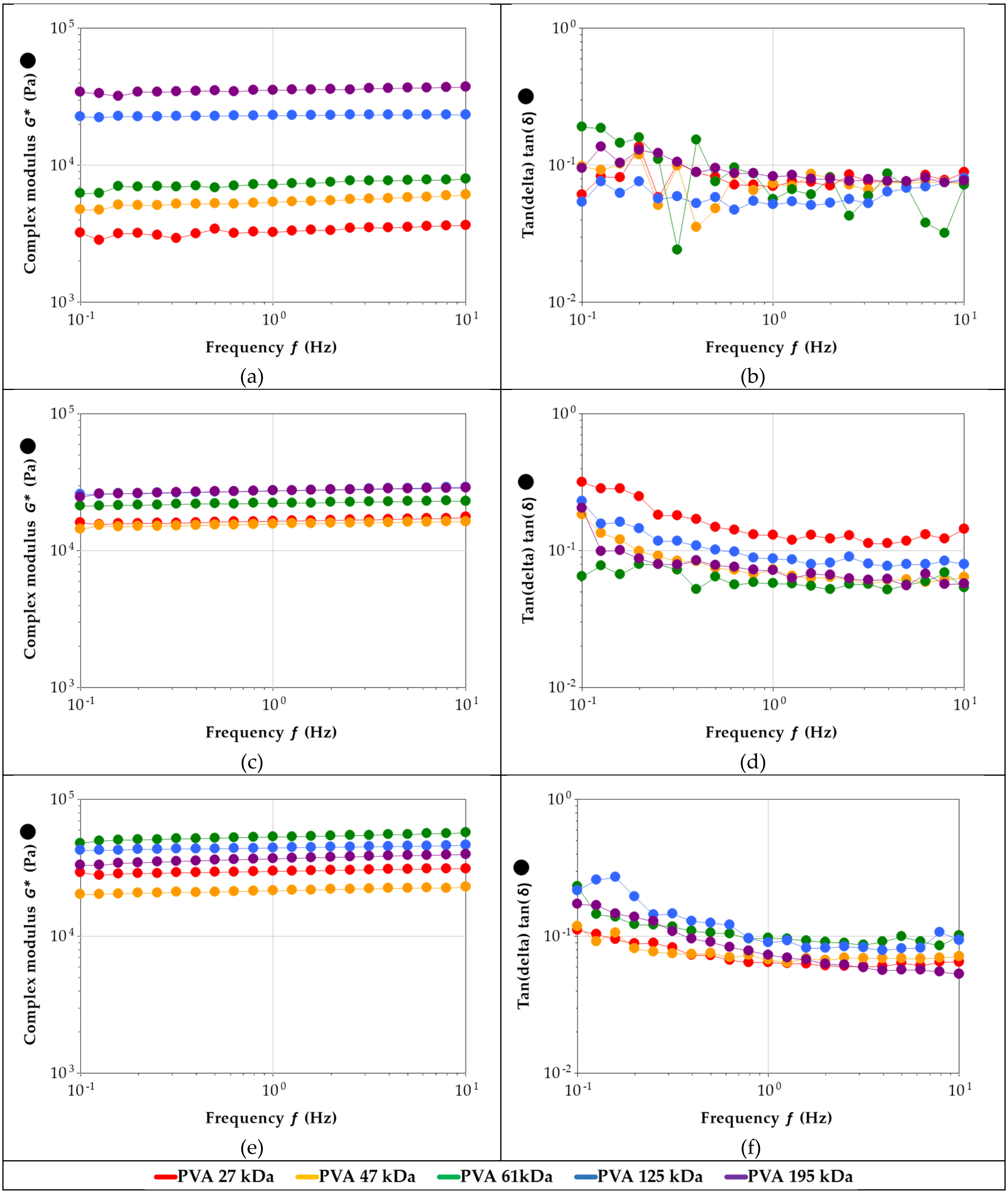

Figure S3: The G* (a,c,e) and the tan δ (b,d,f) resulted from the frequency sweep test for the PEG 4 (a, b), PEG 8 (c, d), PEG 20 (e, f).
